# Embracing Tradition: The Vital Role of Traditional Foods in Achieving Nutrition Security

**DOI:** 10.3390/foods12234220

**Published:** 2023-11-22

**Authors:** Sampat Ghosh, Victor Benno Meyer-Rochow, Chuleui Jung

**Affiliations:** 1 Agriculture Science and Technology Research Institute, Andong National University, Andong 36729, Republic of Korea; sampatghosh.bee@gmail.com; 2 Department of Ecology and Genetics, Oulu University, 90140 Oulu, Finland; meyrow@gmail.com; 3 Department of Plant Medicals, Andong National University, Andong 36729, Republic of Korea

In a press release from the Convention on Biological Diversity (CBD) in 2019, it was reported that, currently, a mere 12 plants and 5 animal species account for approximately 75% of global food production [1]. This heavy reliance on a limited range of high-yielding crop varieties and animal species has led to the gradual neglect of many local food sources, including those involving plants and animals [2,3,4]. To exemplify with a few instances: the prevalence of rice and wheat in Asia can be attributed to their abundant availability, their ease of cultivation, and the influence of global market trends. However, this increasing reliance on these staple crops has resulted in the neglect of traditional crops, such as millet, sorghum, and various local varieties of rice and wheat. This neglect, in turn, leads to the depletion of the unique flavors, nutritional richness, and cultural significance associated with traditional foods [5].

In the Americas, particularly in the United States and South America, corn and soybeans have risen to prominence as dominant crops due to their extensive use in processed foods, livestock feed, and biofuel production. This dominance has fueled the expansion of monoculture farming practices, which can have adverse environmental and health consequences. In contrast, traditional crops, such as quinoa, amaranth, and various indigenous vegetables, are often marginalized in favor of these commodity crops [6,7,8]. The era of globalization has ushered in notable shifts in dietary preferences on a global scale. Among the noteworthy trends attributed to globalization is the movement towards diets centered around a limited selection of crops. This shift carries the risk of diminishing traditional foods and reducing agricultural diversity. Furthermore, the global proliferation of export-oriented agriculture and processed and convenience foods, and the widespread presence of fast-food chains and processed food products have collectively contributed to the erosion of traditional food systems and the loss of culinary heritage on a global scale. Consequently, this trend is causing shifts in dietary patterns, which can have a significant impact on the nutritional well-being of individuals and, on a broader scale, the communities that they belong to. Hence, it is imperative to record the regional cuisines at risk of disappearance.

This Special Issue titled “Traditional and Ethnic Foods in the Context of Food Nutritional Security” features 10 articles and 1 communication. These articles highlight traditional cuisines from various communities; emphasize the significance of traditional wisdom concerning local biodiversity as a means of sustainable food production; and highlight the value of merging traditional foods with advanced scientific techniques to enhance quality and, consequently, bolster nutritional security.

Traditional foods, often rooted in centuries-old culinary traditions, hold a special place in the cultural fabric of societies across the globe. These foods are deeply embedded in the customs, rituals, and histories of communities, which pass down their knowledge from one generation to the next. Traditional foods are often characterized by their diversity, locally adapted ingredients, and unique preparation methods, making them a treasure trove of culinary heritage. In their study, Downs et al. [9] analyzed the value chains of indigenous foods in Jharkhand, India (finger millet for the Munda tribe and Koinaar leaves for the Sauria Paharia tribe). Their findings suggest that interventions such as better storage, processing machinery, and improved cooking fuel for finger millet can slow down or stop the loss of traditional foods, while, for Koinaar leaves, improving drying techniques, language support, and local market access can enhance supply chains and promote consumption among indigenous communities in India.

Traditional foods are not just a reflection of culture; they are frequently also a source of vital nutrients. These foods are rich in vitamins, minerals, antioxidants, and dietary fiber, contributing to overall health and well-being. Moreover, traditional diets have been associated with reduced risks of chronic diseases, such as heart disease, diabetes, and obesity. By incorporating traditional foods into modern diets, we can promote healthier eating patterns and prevent diet-related health problems. Tshikororo et al. [10] focused on investigating the nutritional content of ten wild indigenous vegetables commonly consumed by the Basotho people in southern Africa. These vegetables were *Asclepias multicaulis*, *Lepidium africanum*, *Erucastrum austroafricanum*, *Solanum nigrum*, *Sonchus dregeanus*, *S. integrifolius*, *S. nanus*, *Rorippa fluviatilis*, *Tribulus terrestris*, and *Urtica lobulata*. The research aimed to fill knowledge gaps regarding the nutritional value of these vegetables. The findings revealed that some of these wild vegetables, such as *A. multicaulis* and *S. dregeanus*, are rich in various essential minerals and protein content, making them comparable to commercialized vegetables. Additionally, all of the studied wild vegetables were found to have low levels of cadmium, copper, and lead, making them safe for consumption.

Zou et al. [11] highlight the importance of exploring underutilized food sources beyond the 10 major crops that primarily feed the world. Their study focuses on “Miwu,” the aerial part of the medicinal plant Rhizoma Chuanxiong, as a traditional Chinese local food resource. The research examines Miwu’s historical consumption, chemical components, safety aspects, and current industrial development through textual research, fieldwork, and SWOT analysis. The findings reveal that Miwu has a long history of consumption, is rich in nutrients, lacks acute toxicity, and has potential for further industrial development. The paper offers recommendations for the industrialized development of Miwu, aiming to contribute to the future utilization and development of this underexplored food resource.

Typically, local people with traditional lifestyles incorporate various plants such as leafy vegetables and wild fruits into their planned diets, benefiting from both the nutritional and medicinal aspects of these plants. This dual usage often blurs the distinction between traditional foods and traditional medicines, posing a challenge. Anwar et al. [12] focused on exploring the traditional knowledge and practices of indigenous communities in Bahawalpur and adjacent regions in Pakistan, particularly regarding the use of food as well as ethno-medicinal wild plants to treat various diseases. The research collected and evaluated local knowledge, plant usage, and resource management through in-person interviews and various ethnobotanical analytical techniques. A total of 158 plant species from 49 families were identified, with perennial herbs being the most documented therapeutic plants. The study highlighted the importance of these plants in treating common ailments, emphasizing the need for their conservation and innovative applications to support the local population’s reliance on herbal remedies for healthcare.

Traditional foods are often sourced from local ecosystems and rely on indigenous crops and animal breeds. Preserving traditional food systems promotes biodiversity, safeguards endangered species, and supports sustainable agriculture in the face of unforeseen climate change. Traditional farming practices, passed down through generations, emphasize the importance of harmonious relationships with nature, ensuring that ecosystems remain resilient and productive. Foraging wild mushrooms has a rich history and tradition in Central Europe. The mushrooms serve as a valuable food source due to their nutritional benefits. Wild mushrooms, with their relatively high protein content, have been a traditional meat substitute in European cuisines, particularly during crises such as wars and pandemics [13]. Procházka et al. [13] further demonstrated that wild mushrooms can substitute approximately 0.2% of daily protein intake and contribute about 3% to the agricultural output of the Czech economy, representing Central Europe. The actual calculated price of wild mushrooms suggests their growing popularity as a protein source in Central Europe, with prices seemingly unaffected by supply quantities. 

It is crucial to merge contemporary technological advancements with traditional culinary practices to elevate food quality, ultimately leading to an improvement in the nutritional aspects of diets. Özberk et al. [14] focused on evaluating suitable genotypes for frike production, an ancient and traditional food made from early-harvested whole wheat grain, mainly durum wheat (DW). Frike is considered a functional food with health benefits and is produced in West Asian and North African countries, particularly in Southeastern Turkey. The research assessed 20 DW cultivars and DW-*Thinopyrum ponticum* introgression lines (ILs) based on frike yield, quality, market price, and profitability. The study, conducted in Gölbaşı, Turkey, during the 2021–2022 season, identified Turkish varieties such as Tüten-2002, Edessa, Artuklu, and Perre, along with the R5 IL, as having the highest frike yields. The Turkish varieties Sariçanak-98, Burgos, Sümerli, and Artuklu, along with the R112 IL, excelled in quality and obtained high market prices. Overall, the Turkish cultivars Artuklu, Firat-93, and Sariçanak-98, along with the R112 IL, were identified as the most suitable genotypes for frike production, preserving cultural and genetic diversity in food production from durum wheat.

Ramirez-Jiménez et al. [15] investigated the impact of using ohmic heating (OH) in tortilla production, as an eco-friendly alternative to traditional nixtamalization. Using a rat model, the research compared the nutritional value of tortillas made with OH to that of traditionally processed tortillas (TPTs) and commercial tortillas (CTs). Despite similar protein and macronutrient profiles, OH-processed tortillas had a higher insoluble fiber content and exhibited superior protein digestibility. Although the tortillas had a lower protein content leading to moderate malnutrition indicated by serum albumin in rat, the protein efficiency ratio showed no significant difference from TPTs. Bone characteristics and fracture strength were improved in tortilla-fed groups, and the study suggested that OH could be a sustainable method for tortilla production while maintaining a nutritional value comparable to that of traditional methods. Olusanya et al. [16] discussed the potential of enriching staple foods such as Ujeqe with Amaranthus leaf powder (ALP) to enhance the mineral content to mitigate a malnutrition situation. The study found that ALP supplementation increased protein, ash, and certain mineral content, but higher ALP concentrations led to a lower consumer acceptability of Ujeqe. Further research could explore the fiber content of ALP-supplemented Ujeqe.

The preservation and promotion of traditional foods are intimately linked to cultural preservation and identity. These foods are not just sources of sustenance; they carry stories and memories, and they create a sense of belonging. Reviving and celebrating traditional foods can help communities reconnect with their roots, bolster cultural pride, and strengthen social bonds. Pasqualone et al. [17] examined the physical, chemical, and nutritional characteristics of Somali laxoox and Yemeni lahoh flatbreads, which had not previously been studied or compared. The researchers collected samples from households in Somaliland and found variations in the protein content (12.47–15.94 g/100 g) and lipid content (2.47–4.11 g/100 g), influenced by preparation methods and recipes. The total phenolic compounds also varied (5.02–7.11 mg gallic acid equivalents/g on dry matter). Both types of flatbreads had a porous structure, but the Yemeni flatbreads had a higher cell density. The use of more refined flour resulted in a paler bread. Principal component analysis revealed differences between the Yemeni and Somali flatbreads, indicating some variability within the Somali samples, with two samples forming a distinct subgroup.

Iesa et al. [18] focused on the encapsulation of freeze-dried dewaxed cerumen from *Tetragonula laevicpes*, a substance made by stingless bees with antimicrobial and antioxidant properties. The research investigated various coating materials and carrier ratios to create powdered cerumen, addressing its adhesive nature. The results showed that all carrier matrices had high yields, a low moisture content, and good water dispersibility. The encapsulated cerumen exhibited antioxidant activities ranging from 69% to 80% and retained 46% to 68% of the total phenolic content. The carrier ratio of 5:5 was found to be the most suitable, providing better physical properties and preserving 68% of the polyphenolic activity in the powders.

In addition to the typical topics covered in the publication, Professor Meyer-Rochow [19] shared his perspective on how molecularly engineered plant galls could offer a solution to the global food shortage challenge. The world faces two major problems: global food shortages and a decline in pollinating insects. To address these issues, researchers are exploring the potential of plant galls, which are growths on plants that do not require pollination and can resemble fruits in appearance. The idea is to understand how chemical signals from gall-inducing insects trigger the formation of galls in plants. With this knowledge, scientists hope to bioengineer designer galls of various sizes, colors, and contents for use as food or as sources of medicinally valuable compounds. To achieve this, researchers plan to identify the genes responsible for gall formation through techniques such as RNA sequencing and gene expression analysis. Once identified, these genes can be transferred to other plants, potentially using plasmids or viruses, similar to the techniques used in crop improvement. This research even raises the possibility of producing engineered plant galls through genetic manipulation in plant tissue culture, eliminating the need for insects entirely.

Global food and nutrition security can often be best achieved through the implementation of local food systems. While it might seem counterintuitive, these local solutions can address global challenges effectively. Local food systems tend to be more sustainable and have lower carbon footprints because they promote the use of seasonal, locally-sourced ingredients, reducing the need for long-distance transportation and minimizing food waste. These systems also enhance food resilience and accessibility by catering to the specific needs and preferences of local communities. By strengthening local food production and distribution networks, we can contribute to the broader global goal of food and nutrition security, creating a more sustainable and resilient food system for the future. In a rapidly changing world, where the global population continues to grow and dietary habits evolve, the role of traditional foods in ensuring nutrition security is more critical than ever before. As we grapple with the challenges of food security, sustainability, and health, it is essential to recognize the value of traditional foods in addressing these complex issues [20]. In this Special Issue, we delve into the multifaceted aspects of traditional foods and their profound impact on achieving nutrition security.

In a world grappling with food insecurity, malnutrition, and the adverse effects of climate change, traditional foods offer a path forward. Their resilience, adaptability, and nutrient-rich qualities make them valuable assets in addressing these challenges. Research and innovation can play a pivotal role in harnessing the potential of traditional foods to improve nutrition security.

Therefore, the role of traditional foods in achieving nutrition security cannot be overstated. This is why in this Special Issue we explore the myriad facets of traditional foods and explore their nutritional values and health benefits and examine their role in preserving biodiversity and cultural identity. We also highlight the importance of innovative approaches and collaborations between producers and consumers necessary to promote the integration of traditional foods into modern diets. As we navigate the complex landscape of global food systems, it is imperative that we recognize and embrace the wisdom of our food traditions. Traditional foods offer a sustainable, nutritious, and culturally rich path towards a future where everyone has access to the nourishment that they need for a healthy and fulfilling life.
